# A salivary EF-hand calcium-binding protein of the brown planthopper *Nilaparvata lugens* functions as an effector for defense responses in rice

**DOI:** 10.1038/srep40498

**Published:** 2017-01-18

**Authors:** Wenfeng Ye, Haixin Yu, Yukun Jian, Jiamei Zeng, Rui Ji, Hongdan Chen, Yonggen Lou

**Affiliations:** 1 State Key Laboratory of Rice Biology, Institute of Insect Science, Zhejiang University, Hangzhou 310058, China

## Abstract

The brown planthopper (BPH), *Nilaparvata lugens* (Stål) (Hemiptera: Delphacidae), a major pest of rice in Asia, is able to successfully puncture sieve tubes in rice with its piercing stylet and then to ingest phloem sap. How BPH manages to continuously feed on rice remains unclear. Here, we cloned the gene *NlSEF1*, which is highly expressed in the salivary glands of BPH. The NlSEF1 protein has EF-hand Ca^2+^-binding activity and can be secreted into rice plants when BPH feed. Infestation of rice by BPH nymphs whose *NlSEF1* was knocked down elicited higher levels of Ca^2+^ and H_2_O_2_ but not jasmonic acid, jasmonoyl-isoleucine (JA-Ile) and SA in rice than did infestation by control nymphs; Consistently, wounding plus the recombination protein *NlSEF1* suppressed the production of H_2_O_2_ in rice. Bioassays revealed that *NlSEF1*-knockdown BPH nymphs had a higher mortality rate and lower feeding capacity on rice than control nymphs. These results indicate that the salivary protein in BPH, NlSEF1, functions as an effector and plays important roles in interactions between BPH and rice by mediating the plant’s defense responses.

In nature, plants are constantly threatened by herbivorous insects. Consequently, plants have evolved both constitutive and induced defenses that appear after herbivore attack^1^. Inducible defenses begin with the recognition of specific herbivore-associated molecular patterns (HAMPs) and are followed by the elicitation of a complex signaling network, consisting mainly of mitogen-activated protein kinase (MAPK) cascades, and jasmonic acid (JA), salicylic acid (SA), and ethylene (ET) signaling pathways, which subsequently results in the reconfiguration of the transcriptome and proteasome as well as the biosynthesis of defensive chemicals^2^,^3^. This HAMP-triggered immunity (HTI) is effective with some herbivore populations but not with herbivores that secrete effectors which can effectively suppress the HTI^3^. In that case, plant genotypes that contain resistance genes can recognize these effectors and thereby result in effective and specific effector-triggered immunity^3^,^4^. Thus, HAMPs and effectors derived from herbivore saliva and egg secretions play important roles in plant–herbivore interactions^3^.

Thus far, the chemicals in many HAMPs from herbivore saliva and egg secretions have been identified, and these include proteins, peptides, lipids and other small molecule compounds, such as fatty acid–amino acid conjugates, β-glucosidase, inceptins and caeliferins^2^,^4^. Moreover, the mechanisms underlying HAMP-elicited defense responses have also been extensively studied^4^,^5^. In contrast, herbivore effectors have been less well studied. It has been reported that some salivary proteins from herbivores, such as glucose oxidase from *Helicoverpa zea* and C002, Mp10, Me10, Me23 from aphids^6^,^7^, function as effectors of plant defense. These effectors also include salivary calcium binding proteins, which can bind to Ca^2+^ and thus suppress a plant’s defenses. For example, saliva secreted by the aphid *Megoura viciae* contains Ca^2+^-binding proteins which can bind to Ca^2+^ and then lead to the contraction of forisomes; such contraction prevents the plugging of sieve elements and facilitates an aphid’s continuous ingestion from sieve tubes^7^,^8^. The green rice leafhopper *Nephotettix cincticeps* has been observed to secrete an 84-kDa salivary EF-hand Ca^2+^-binding protein into sieve tubes during leafhopper feeding; this protein may also suppress the clogging of the tube^9^. However, the molecular mechanism of effector-mediated suppression of plant defenses remains largely unknown^10^.

The calcium ion (Ca^2+^) is a ubiquitous and essential secondary messenger in all eukaryotic organisms and plays an important role in all aspects of cell function, including mediating responses to various biotic and abiotic environmental cues^11^,^12^. Calcium-binding proteins, which are a component of the calcium-signaling pathway, are calcium sensors; they decode the information presented in Ca^2+^ signatures. Characterized by their amplitude, duration, frequency, and location, these proteins relay the Ca^2+^ signal to specific downstream responses^12^. The majority of Ca^2+^-binding proteins belong to the EF-hand proteins, which contain a motif characterized by a helix–loop–helix structure, with an interhelical loop of 11 to 14 amino acids that can bind to Ca^2+^ ion^13^. In plants, the major family of EF-hand Ca^2+^-binding proteins includes calmodulin (CaM), calmodulin-like proteins (CMLs), calcium-dependent protein kinases (CDPKs) and calcineurin B-like proteins (CBLs)^14^,^15^. It has been well documented that the calcium-signaling pathway plays a central role in plant defense against pathogens^12^,^15^. Ca^2+^ elevation, for instance, which was reported to be one of the earliest signaling events in plants after the perception of pathogen-associated molecular patterns, is crucial for downstream responses in plant-pathogen recognition^16^. Recently, the calcium-signaling pathway has also been found to be involved in plant defense against herbivores. Herbivore infestation and the application of herbivore oral secretions or HAMPs were observed to induce Ca^2+^ transients^17^. In addition to the role played by calcium signaling in the plugging of sieve tubes as mentioned above, several CDPKs^18^,^19^ and CMLs^20^,^21^ have been reported to regulate defense-related signaling pathways and defense responses in plants.

The rice brown planthopper (*Nilaparvata lugens* (Stål), BPH) is one of the most economically devastating pests in the world. In rice plants, BPH reduces the photosynthetic rate, leaf and stem nitrogen concentrations, chlorophyll content, and organic dry weight, thereby dramatically decreasing yield. The cultivation of resistant varieties of rice is the most effective and environmentally friendly way to control BPH. However, BPH rapidly overcomes rice resistance by evolving new virulent populations^22^. Although some minor differences in morphology and in the composition of bacterial symbionts among virulent genotypes have been reported, the mechanisms underlying BPH virulence are not clear^23^,^24^. As a phloem-feeding herbivore, BPH secretes two primary kinds of saliva: coagulable and watery. By forming salivary sheaths, the coagulable saliva helps to stabilize the insects’ stylets and to suppress plant defense responses to components of the watery saliva^23^,^25^. The watery saliva, which contains a mixture of digestive enzymes, such as alkaline phosphatase, esterase, amylase, and β-glucosidase, as well as other components such as proteins related to pathogen transmission^6^,^23^, assists the movement of the stylets inside the salivary sheath, the digestion of plant materials, and the modulation of plant defense responses^6^,^25^. Therefore, BPH saliva plays an important role in interactions between BPH and its host plants. Up to now, salivary proteins, expressed sequence tags and the transcriptome of the salivary glands of BPH have been analyzed^23^,^26^,^27^,^28^. Moreover, several salivary proteins, such as a catalase-like protein Kat-1, salivap-3, NlShp and annexin-like5, have been found to play a role in the formation of salivary sheath and/or BPH feeding^26^,^27^,^29^. However, whether these putative salivary proteins influence plant defense and thus mediate the feeding ability of BPH remains unclear.

By screening all of 352 reported genes encoding putative secreted proteins of salivary gland of BPH^23^, we identified 6 genes encoding proteins with EF-hand calcium binding domains. Given the important role of salivary EF-hand proteins in plant defenses stated above, we thus investigated the effect of these 6 genes on survival rates of BPH nymphs after one of these genes was knocked down by RNA interference (RNAi). Of these genes, *NlSEF1* was found to have an obvious effect on the survival of BPH nymphs on rice (see below). Therefore, in this study, we chose *NlSEF1* and explored its role in rice-BPH interactions. By combining molecular biology, chemical analysis and bioassays, we suggest that NlSEF1 is an herbivore effector that suppresses the host plant’s defense responses and thus enhances the virulence of BPH.

## Results

### Isolation and Characterization of *NlSEF1*

**Figure 1:**
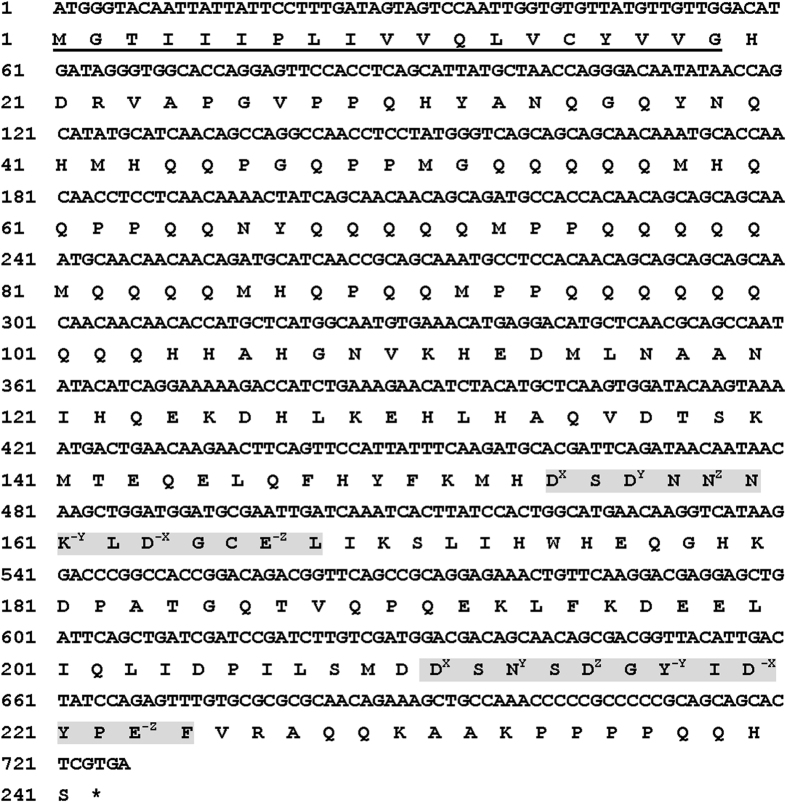
Nucleotide sequence of *NlSEF1* and its deduced amino acid sequence. The predicted signal peptide is underlined, and the terminal codon is marked with an asterisk (*). Sequences of the two EF-hand domains are boxed in light gray shading. Residues involve in Ca^2+^-binding are noted by superscripts, which represent the position of residues in the loop.

Based on the data from transcriptomes of BPH salivary glands^23^, the open reading frame (ORF) (726 bp) of the gene *NlSEF1* was obtained by reverse-transcription-polymerase chain reaction (RT-PCR) (Fig. 1, GenBank: KT698079). Sequence analysis revealed that *NlSEF1* encodes a 241-amino-acid protein with a predicted molecular weight of 28.06 kDa and a pI of 5.93. The protein possesses an extracellular signal peptide and has no transmembrane domains, suggesting that NlSEF1 is a putative secreted protein. The mass of the predicted mature protein is 26.04 kDa and the protein has a pI of 5.94. There was no potential O-glycosylation or N-glycosylation site in the protein. The predicted secondary structure of NlSEF1 contained eight coil regions, seven α-helixes and no β-sheet (Supplemental Fig. S1). The PROSITE scan indicated two EF-hand calcium-binding domains (EF_HAND_1, PS00018) in the C-terminus of NlSEF1. Both domains contained a 12-residue loop that begins with aspartic acid and ends with glutamic acid, and each loop was surrounded by two α-helixes (Supplemental Fig. S1), showing a canonical helix-loop-helix EF-hand motif.

Protein alignment revealed that NlSEF1 is homologous to a predicted multiple coagulation factor deficiency protein 2 (MCFD2) that belongs to the EF-hand 7 family (PF13499) and is characterized by a pair of EF-hand domains. In humans, MCFD2 is a soluble luminal protein and a part of a cargo-specific endoplasmic reticulum-to-Golgi transport complex^30^. However, no evidence shows that homologs in insects play similar roles. NlSEF1 has the highest identity to a predicted MCFD2 of *Pediculus humanus corporis* (XP_002429496.1, 77% identity) but very low query coverage (46%). The best blast hit is a predicted MCFD2 of *Solenopsis invicta* (XP_011158313.1) when sorted by max or total blast score (96% coverage, 55% identity). Unlike the high similarity of C-terminals, the similarity of N-terminals is low except the 11 amino acid residues after the signal peptide (Supplemental Fig. S2).

**Figure 2:**
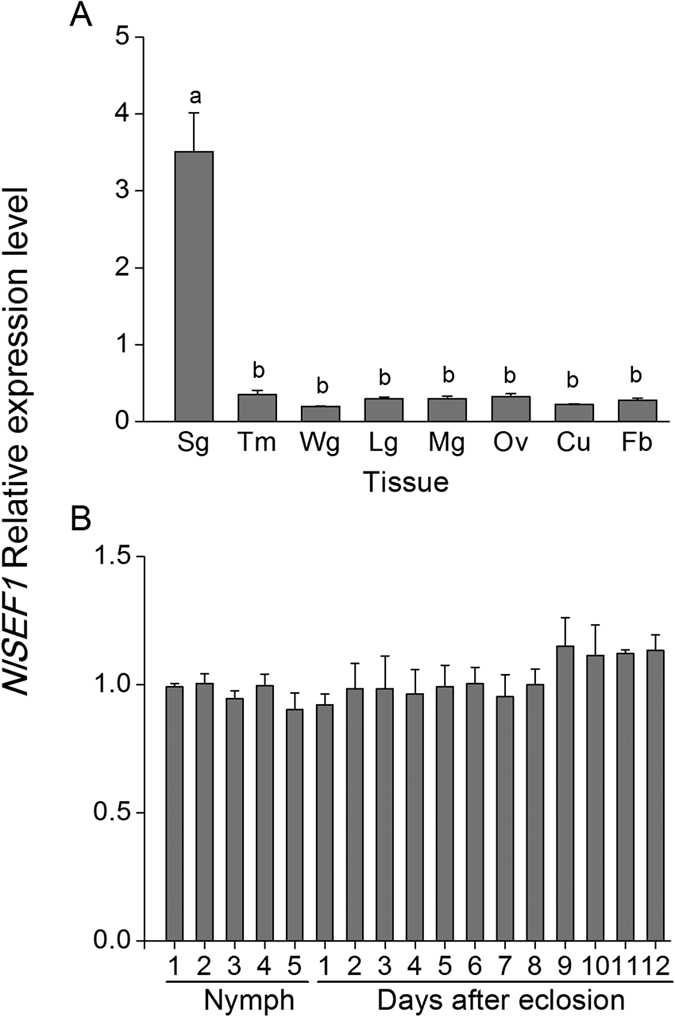
Mean transcript levels (+SE, *n* = 3) of *NlSEF1* in different tissues (**A**) and developmental stages (**B**) of BPH. Sg, salivary gland; Tm, thorax muscle; Wg, wing; Lg, leg; Mg, midgut; Ov, ovary; Cu, cuticle; Fb, fat body. Nymph 1–5, first- to fifth-instar larvae; days after eclosion 1–12, 1- to 12-d-old-female adult. Letters indicate significant differences among different treatments (p < 0.05, Duncan’s multiple range test).

*NlSEF1* was highly expressed in the salivary glands, but low expression levels (approximately 10-fold lower) were detected in other tissues, including wing, leg, thorax muscle, midgut, cuticle, ovary, and fat body (Fig. 2A). The expression level of *NlSEF1* at different BPH developmental stages was constant and only slightly higher in older (9- to 12-d-old) female adults (Fig. 2B).

**Figure 3:**
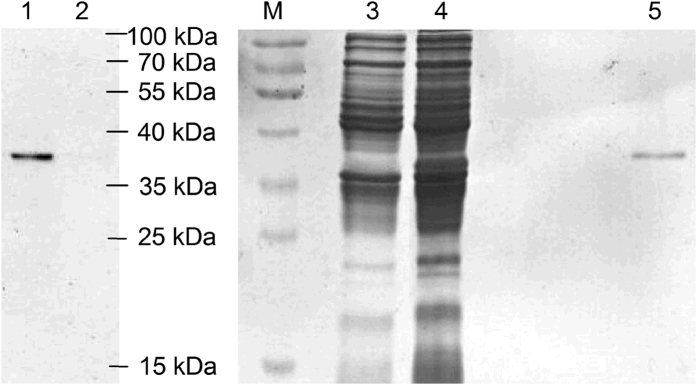
Expression and purification of NlSEF1. Samples for Western blot (lanes 1 and 2) and SDS-PAGE analysis (lanes 3 to 5) were as follows: concentrated supernatant from *Escherichia coli* with the empty vector pET-30a (lanes 2 and 3; control); concentrated supernatant from *E. coli* with the recombinant vector *NlSEF1*:pET-30a (lanes 1 and 4); purified recombinant protein NlSEF1 (lane 5) and protein marker (M). Rabbit anti-NlSEF1 polyclonal antibodies were used to develop Western blot.

**Figure 4:**
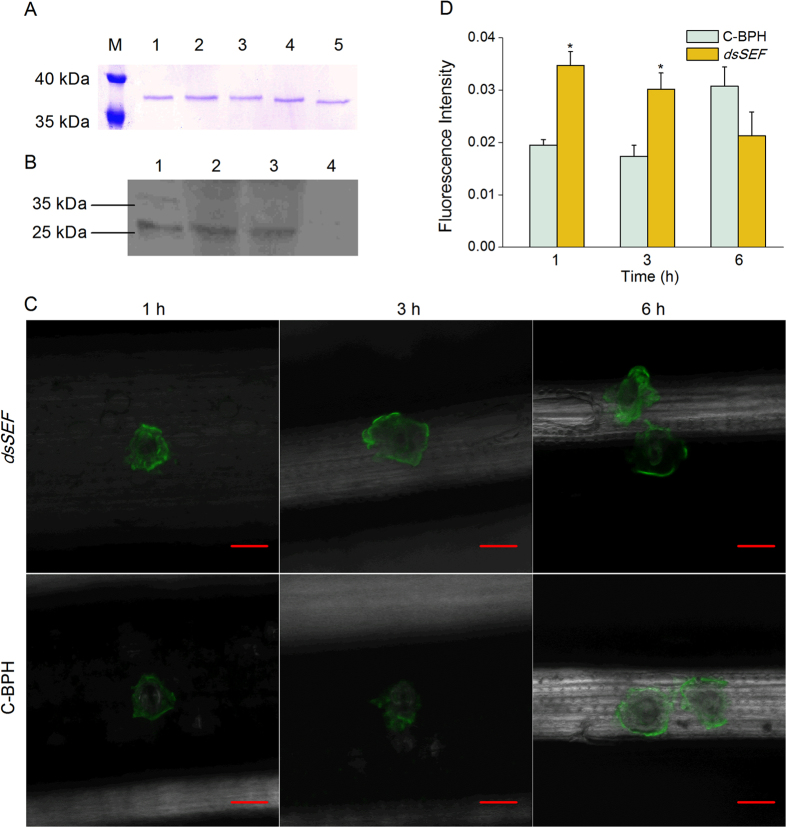
NlSEF1 in rice and its molecular characterization. (**A**) Ca^2+^-dependent mobility of purified NlSEF1 under SDS-PAGE conditions. The NlSEF1 protein was incubated for 30 min at 25 °C with each of the following solutions: lane 1, 0.5 mM CaCl_2_; lane 2, 0.15 mM CaCl_2_; lane 3, 0.05 mM CaCl_2_; lane 4, 0.015 CaCl_2_; lane 5, 0.5 mM EDTA. (**B**) Western blot analysis of protein NlSEF1 in rice infested by BPH nymphs. Lane 1, the extracts from the salivary glands of BPH; lanes 2–4, the extracts from rice plants of Mudgo that were infested by nymphs (lanes 2 and 3) or kept non-infested (lane 4). (**C**) Fluochemical intracellular Ca^2+^ determination in leaves infested by BPH. The green fluorescence refers to binding of Fluo-3 AM with Ca^2+^. A portion of rice leaf incubated with 5 μM Fluo-3 AM solution that was infested by newly emerged BPH female adults which had been injected with the dsRNA of *NlSEF1* (dsSEF-BPH, *dsSEF*) or kept non-manipulated (C-BPH) for 1, 3 and 6 h. Metric bar = 25 μm. (**D**) Mean fluorescence intensity at feeding sites of newly emerged female adults of dsSEF-BPH (*dsSEF*) or C-BPH. Asterisks indicate significant difference between treatments (P < 0.05, t-test).

Recombinant protein NlSEF1 (with a mass of about 37 kDa) was produced in a bacterial expression system (Fig. 3). The Ca^2+^-binding capability of NlSEF1 was verified by a gel mobility shift assay. Purified NlSEF1 mixed with different concentrations of CaCl_2_ was subjected to SDS-PAGE. Compared with the mobility of NlSEF1 in the presence of 0.5 mM EDTA, the mobility of NlSEF1 was slowed by the addition of 0.015 to 0.5 mM CaCl_2_. The migration of NlSEF1 appeared to slow when the concentration of CaCl_2_ was high (Fig. 4A), suggesting that NlSEF1 has the Ca^2+^-binding capability.

### NlEG1 Is a Secreted Protein that Enters Rice Plants When BPH Feeds

Western blot analysis with NlSEF1 antibodies was performed to verify whether the protein was secreted into the rice plants. Approximately 200 fifth-instar nymphs of BPH were placed on individual rice plants for 24 h. The total proteins from plant stems were extracted and Western blot analysis was performed using polyclonal anti-NlSEF1 rabbit antibodies. A band of about 26 kDa was detected in extracts from BPH salivary glands (Fig. 4B; lane 1). The same band was also detected in plants infested by BPH (Fig. 4B; lanes 2 and 3). In contrast, no NlSEF1 band was detected in plants that had not been exposed to BPH (Fig. 4B; lane 4). These results suggested that NlSEF1 was injected as a salivary component into the rice plants when BPH fed.

### NlSEF1 Decreases the Levels of Cytosolic Ca^2+^ in Rice

Since NlSEF1 has the Ca^2+^-binding capability, we were interested in whether NlSEF1 could influence the cytosolic Ca^2+^ content, [Ca^2+^]_cyt_, in rice plants. To investigate this issue, we used RNAi as described in Liu *et al*.^31^ to obtain a BPH population in which *NlSEF1* had been silenced (dsSEF-BPH) and analyzed [Ca^2+^]_cyt_ in leaves of plants that were infested with dsSEF-BPH or BPH that were kept non-manipulated (C-BPH). Injecting BPH with the dsRNA decreased the transcript levels of *NlSEF1* in the whole body and salivary gland of the insect by 62–88% over a period of 10 days (Supplemental Fig. S3). Silencing NlSEF1 in BPH did not influence the growth phenotype of individuals (Supplemental Fig. S4). The [Ca^2+^]_cyt_ was investigated by confocal laser scanning microscopy using Fluo-3 AM (acetoxy-methyl ester of Fluo-3) as the Ca^2+^-selective fluorescent indicator. Clear fluorescence was observed around BPH feeding sites (Fig. 4C), suggesting that BPH feeding activates cytosolic Ca^2+^ transient in rice. Moreover, fluorescence intensity around feeding sites by dsSEF-BPH was significantly higher than that around feeding sites by C-BPH at 1 and 3 h but not 6 h after BPH infestation (Fig. 4C and D); this demonstrates that the [Ca^2+^]_cyt_ at dsSEF-BPH feeding sites was higher than those at C-BPH feeding sites. These results suggested that the Ca^2+^-binding protein NlSEF1 from BPH seems to be the determinant of [Ca^2+^]_cyt_ in plants when they were infested by BPH.

### NlSEF1 Suppresses the Production of H_2_O_2_ in Rice

**Figure 5:**
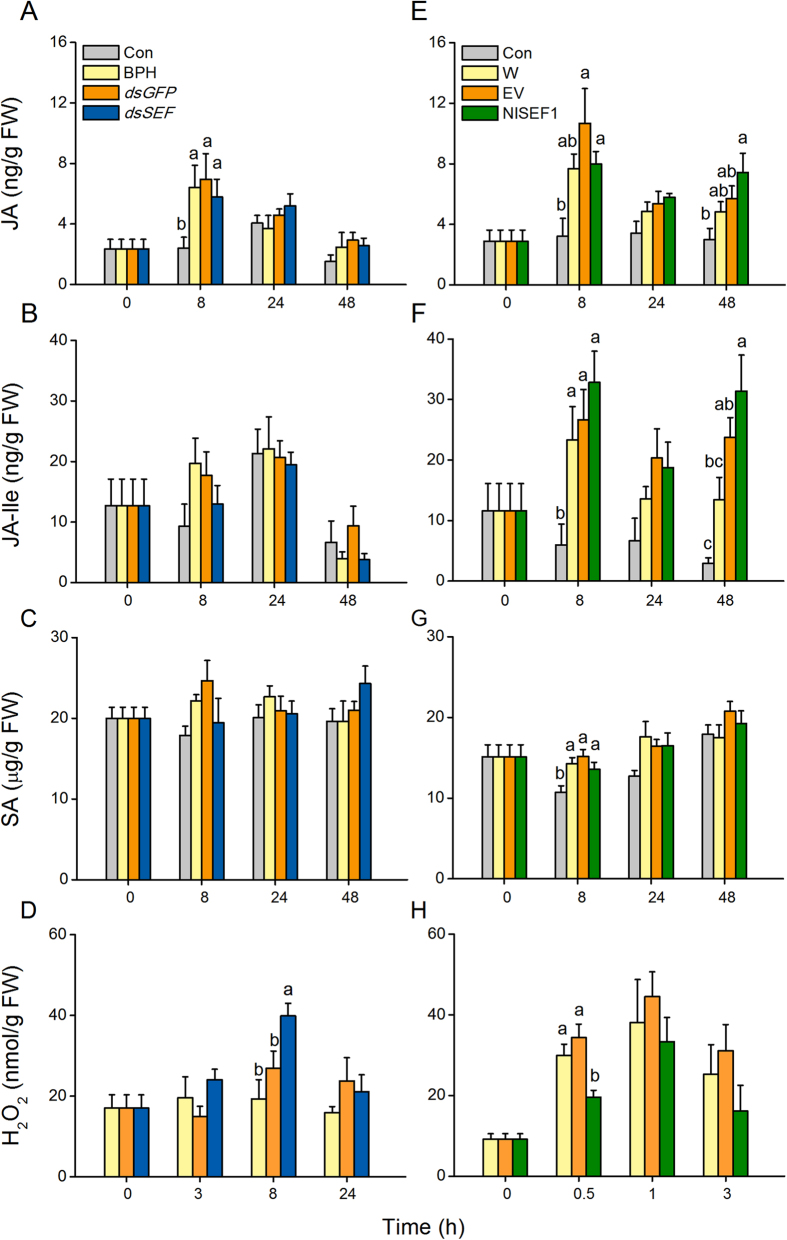
Effect of NlSEF1 protein on jasmonic acid (JA), jasmonoyl-isoleucine (JA-Ile), salicylic acid (SA) and hydrogen peroxide (H_2_O_2_) contents in rice plants. (**A** to **D**), Mean levels (+SE, *n* = 5) of JA (**A**), JA-Ile (**B**), SA (**C**) and H_2_O_2_ (**D**) in plants that were kept non-manipulated (Con) or infested by fifth-instar nymphs that had been injected with the dsRNA of *NlSEF1 (dsSEF*) or GFP (*dsGFP*), or kept non-manipulated (C-BPH). (**E** to **H**) Mean levels (+SE, *n* = 5) of JA (**E**), JA-Ile (**F**), SA (**G**) and H_2_O_2_ (**H**) in plants that were kept non-manipulated (Con) or treated with wounding plus the recombinant protein NlSEF1 (NlSEF1), the purified products of the empty vector (EV) or nothing (Wound). Letters indicate significant differences among different treatments (*P* < 0.05, Duncan’s multiple range test).

The JA, JA-Ile, SA, and H_2_O_2_ signaling pathways are reported to play central roles in plant defense responses in many plant species, including rice^32^,^33^,^34^,^35^. Therefore, we asked whether NlSEF1 influenced the biosynthesis of these phytohormones and signals. Our results revealed that C-BPH nymph infestation did not induce the production of these defense-related phytohormones and signals, except the case of JA whose levels were enhanced 8 h after infestation (Fig. 5A–D), whereas mechanical wounding enhanced levels of these phytohormones and signals (Fig. 5E–H). The levels of JA, JA-Ile and SA were similar among rice plants infested with dsSEF-BPH, BPH that had been injected with the dsRNA of *GFP* (dsGFP-BPH) and C-BPH (Fig. 5A–C), whereas H_2_O_2_ levels were significantly higher in the dsSEF-BPH-infested plants than in the dsGFP-BPH- and C-BPH-infested plants at 8 h (Fig. 5D). Consistently, the exogenous application of the recombinant protein NlSEF1 on plants, compared to mechanical wounding and the exogenous application of the purified products of the empty vector, had no influence on JA, JA-Ile and SA biosynthesis, but suppressed the production of H_2_O_2_ 0.5 h after the start of the treatment (Fig. 5E–H). Together, our data demonstrate that the NlSEF1 of BPH is likely to modulate H_2_O_2_ levels in rice plants.

### Knockdown of *NlSEF1* Decreases the Feeding Capacity but Increases Mortality of BPH Nymphs

**Figure 6:**
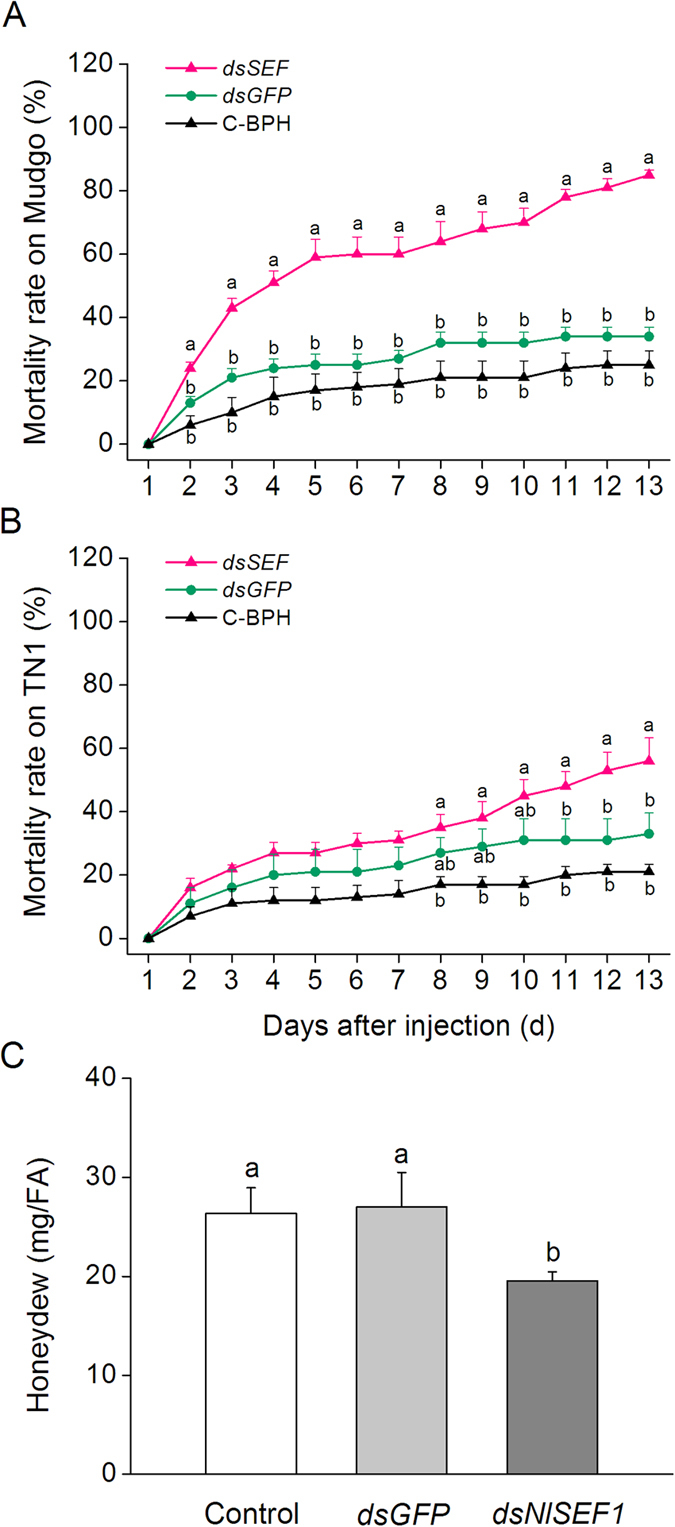
The knockdown of *NlSEF1* increases the mortality rates of BPH nymphs and reduces the honeydew production of female BPH adults. (**A** and **B**) Mean mortality rates (+SE, n = 5) of BPH nymphs which had been injected with the dsRNA of *NlSEF1 (dsSEF*) or *GFP (dsGFP*), or kept non-manipulated (C-BPH) at the third-instar nymph stage, feeding on variety Mudgo (**A**) or TN1 (**B**). (**C**) Mean amount of honeydew per day (+SE, n = 40) secreted by a BPH female adult that had been injected with the dsRNA of *NlSEF1 (dsSEF*) or *GFP (dsGFP*) at the fifth-instar nymph stage or kept non-manipulated (C-BPH). Letters indicate significant differences among different treatments (*P* < 0.05, Duncan’s multiple range test).

We investigated whether NlSEF1 could influence the feeding capacity and mortality rate of BPH and whether its effects changed with the rice varieties on which BPH fed. Mortality among BPH fed on rice varied: compared with dsGFP-BPH and C-BPH, dsSEF-BPH had a significantly higher mortality rate over 1 to 12 days, and dsSEF-BPH fed on the variety Mudgo than on the susceptible variety TN1 had higher mortality rates (Fig. 6). Moreover, the amounts of honeydew, an index for food intake, secreted by dsSEF-BPH were significantly lower than those secreted by dsGFP-BPH and C-BPH, whereas there was no significant difference in the amounts of honeydew between the dsGFP-BPH and C-BPH (Fig. 6C). These results suggest that NlSEF1 is essential to the feeding and survival of BPH.

## Discussion

So far, several salivary Ca^2+^-binding proteins in insects, such as NcSP84^9^ and Armet^36^, have been reported. Some of these proteins can bind Ca^2+^ and then decrease host defense by preventing sieve tube plugging^3^. Here we found that *NlSEF1* is most highly expressed in BPH salivary glands and its mRNA level remains the same in immature and mature stages of BPH (Fig. 2). Moreover, NlSEF1, containing two EF-hand motifs in its C-terminus, can be injected into rice plants when BPH are feeding (Fig. 4B). This result implies that NlSEF1 may play an important role in rice-BPH interactions. Indeed, the mobility of NlSEF1 on the SDS-PAGE gel was gradually reduced by increasing the concentration of calcium from 0.015 to 5 mM (Fig. 4A). Moreover, rice plants infested by dsSEF-BPH had higher cytoplasmic Ca^2+^ contents than did plants infested by C-BPH at 1 and 3 h after infestation (Fig. 4C,D). These data suggest that BPH secrete NlSEF1, a salivary protein which can regulate calcium signaling pathway in rice by binding to cytosolic Ca^2+^. Interestingly, the observed molecular mass (about 37 kDa) of the purified recombinant NlSEF1, using the *E. coli* expression system, was about 11 kDa bigger than the predicted mass of mature NlSEF1, 26.04 kDa. The mass may be explained by the 51 additional amino acids from the expression vector (causing the mass of the recombinant protein to increase to 31.63 kDa) and strong positive charge of the His-tag. Some His-tag fusion proteins have been reported to show higher apparent molecular weight in SDS-PAGE^37^,^38^.

We investigated the influence of NlSEF1 on defense-related signaling pathways in rice and found that rice plants infested by dsSEF-BPH had higher H_2_O_2_ levels than those infested by dsGFP-BPH or C-BPH (Fig. 5D). Consistently, the exogenous application of recombinant NlSEF1 on rice plants decreased the levels of H_2_O_2_ (Fig. 5H). These findings suggest that NlSEF1 secreted by BPH saliva can modulate the H_2_O_2_-mediated signaling pathways in rice plants. Ca^2+^ transients in plants elicited by herbivore infestation are well known to directly or indirectly regulate diverse signaling pathways, such as those mediated by JA, SA, reactive oxygen species (ROS) and NO, which subsequently regulate plant defenses^39^,^40^,^41^. AtSR1, a CaM-binding protein, for example, has been reported to negatively regulate the production of SA by suppressing the expression of EDS1, a positive modulator of SA biosynthesis^42^. In *Solanum tuberosum*, CDPK4 and CDPK5 have been reported to regulate the pathogen-induced ROS production^43^. Moreover, in Arabidopsis, CDPK4, CDPK5, CDPK6 and CDPK11 were found to be involved in ROS production^44^. In rice, BPH infestation has been observed to cause a Ca^2+^ flux and induce the accumulation of JA, SA, ethylene and H_2_O_2_^33^,^34^,^45^,^46^. Therefore, the regulatory effect of NlSEF1 on the level of H_2_O_2_ in rice (Fig. 5) is probably due to its Ca^2+^-binding activity. Further researches should elucidate how NlSEF1-mediated the change in Ca^2+^ levels regulates the biosynthesis of H_2_O_2_ and why this change does not influence the production of other defense-related signals.

Bioassays showed that the knockdown of *NlSEF1* significantly reduced the amount of honeydew secreted by female BPH adults (Fig. 6C). Moreover, when BPH fed on the resistance variety Mudgo, the mortality rate of dsSEF-BPH was higher than that of dsGFP-BPH, whereas, when fed the susceptible variety TN1, the mortality rate of dsSEF-BPH did not differ from that of dsGFP-BPH until the last three days of the experiment (Fig. 6). Our data suggest that NlSEF1 mediates the mortality rate and feeding of BPH not by influencing the insect but by suppressing defense in the plant, suggesting that the protein plays an effector role in rice defense responses. It has been reported that H_2_O_2_ signaling pathways positively regulate resistance in rice to BPH, whereas the JA signaling pathway negatively mediates BPH resistance^32^,^35^. Our experiments showed that rice plants infested by BPH with the knockdown of *NlSEF1* had higher H_2_O_2_ level than those infested by dsGFP-BPH or C-BPH. Thus, the lower performance of dsSEF-BPH on rice plants, compared with the performance of dsGFP-BPH and C-BPH, might be related to the low activity of NlSEF1 in dsSEF-BPH; low activity of NlSEF1 resulted in a high level of H_2_O_2_ in rice plants and thus enhanced the resistance of rice to BPH. Phloem plugging, including callose deposition, which can be induced by Ca^2+^, was also reported to be one of the mechanisms that rice plants use to defend themselves against BPH^47^. Given that rice plants infested by dsSEF-BPH had higher cytoplasmic Ca^2+^ levels than did plants infested by C-BPH (Fig. 4C), it is reasonable to think that phloem plugging might also cause dsSEF-BPH to perform poorly. Further research should elucidate this question.

In summary, NlSEF1 is a salivary EF-hand Ca^2+^-binding protein that can be secreted into rice plants when BPH feed. NlSEF1 binds to Ca^2+^ and then causes a decrease in the content of cytosolic Ca^2+^ in rice; this decrease subsequently reduces H_2_O_2_ levels and possible phloem plugging, which finally impairs resistance in rice to BPH. These findings suggest that the secreted salivary protein NlSEF1 functions as an effector, suppressing defense response in rice plants and allowing BPH to continuously suck the juice from rice phloem.

## Methods

### Plant Growth and Insects

Taichun Native 1 (TN1), a rice variety susceptible to BPH, and Mudgo, a variety containing the resistance gene *Bph1*, were used for experiments. Plants were grown as described by Lu *et al*.^33^, and 35- to 40-day-old plants individually planted in 500 ml hydroponic plastic pots were used. A BPH population – M-BPH – was maintained on Mudgo for more than 170 generations at 27 ± 1 °C and 70 ± 10% relative humidity under a 14/10 h light/dark photoperiod. Insects were originally provided by the Chinese National Rice Research Institute (Hangzhou, China).

### Cloning and Sequence Analysis of *NlSEF1*

The cDNA encoding protein NlSEF1 was obtained by RT-PCR from total RNA isolated from salivary glands of adult BPH females. The primers (Supplemental Table S1) were designed based on the transcriptome data of BPH salivary glands^23^. The PCR products were cloned into the pMD19-T vector (TaKaRa, http://www.takara-bio.com/) and sequenced. The open reading frame (ORF) of *NlSEF1* was predicted using the ORF Finder (http://www.ncbi.nlm.nih.gov/projects/gorf/). The molecular weight and isoelectric point (pI) of the predicted protein were determined using Compute pI/Mw software (http://web.expasy.org/compute_pi/). The signal peptides and transmembrane helices were predicted by using SignalP 4.1 (http://www.cbs.dtu.dk/services/SignalP/) and TMHMM 2.0, respectively. NetNGlyc 1.0 (http://www.cbs.dtu.dk/NetNGlyc) and NetOGlys 4.0 (http://www.cbs.dtu.dk/services/NetOGlyc/) were used to predict N- and O-glycosylation sites, respectively. Secondary structure was predicted by using PSIPRED 3.3 (http://bioinf.cs.ucl.ac.uk/psipred/). Domains of NlSEF1 were searched using PROSITE (http://prosite.expasy.org/). Sequence similarity was searched by using the Blast programme (http://blast.ncbi.nlm.nih.gov/Blast.cgi). Similar amino acid sequences of NlSEF1 downloaded from NCBI (National Center for Biotechnology Information, http://www.ncbi.nlm.nih.gov) were aligned using ClustalW2 (www.ebi.ac.uk/Tools/msa/clustalw2).

### Tissue-specific and Developmental Stage Expression Patterns of *NlSEF1*

Total RNA was extracted from the following materials: (1) eight different BPH tissue samples (salivary gland, wing, leg, thorax muscle, midgut, cuticle, ovary, and fat body) isolated from newly emerged brachypterous female adults; (2) whole bodies of BPH at different developmental stages (from first- to fifth-instar nymphs, newly emerged to 12-d-old brachypterous females). Total RNA was isolated using the SV Total RNA Isolation System (Promega) according to the manufacturer’s instructions. Each RNA sample was reverse-transcribed into cDNA using the Takara Primescript™ RT reagent kit. The QPCR assay was performed on CFX96^TM^ Real-Time system (Bio-Rad, Hercules, CA, USA) using iQ SYBRGreen Supermix (Bio-Rad). A BPH 18S rRNA gene (GenBank accession no. JN662398) was used as an internal standard to normalize cDNA concentrations. The primers used for qRT-PCR are listed in Supplemental Table S1. Three independent biological replicates were analyzed in each experiment.

### Expression of NlSEF1 in *Escherichia coli*

The ORF of *NlSEF1* was amplified by PCR using the primers listed in Supplemental Table S1. The PCR product was ligated into the pMD19-T vector, sequenced, and then cloned into vector pET-30a. The recombinant vector *NlSEF1*:pET-30a and empty vector pET-30a (used as a control) were transformed into *E. coli* BL21 (DE3) strain. Expression was induced by adding IPTG (1 mM final concentration). The products from recombinant and empty vector were purified by using Ni-NTA columns (Qiagen, Venlo, Netherlands) according to the manufacturer’s instructions. The purified products were concentrated with a YM-10 Centricon membrane (Millipore, Billerica, MA, USA) to remove imidazole. The final purified concentrated products from *E. coli* cells with the empty vector and recombinant vector were mixed with 2× SDS loading buffer, respectively, separated by SDS/PAGE in a 12.5% acrylamide gel (ISC Bioexpress, Kaysville, UT, USA), and stained with 0.025% Coomassie blue R-250 in water. The predicted mass of the mature recombinant protein NlSEF1 including 6 N-terminal His-tags is 31.63 kDa.

### Polyclonal Antibody Preparation and Western Blot Analysis

A polypeptide (QQKAAKPPPPQQHS) of NlSEF1 was selected as the antigen, and the polyclonal rabbit antibodies of NlSEF1 were made and purified by GenScript^TM^. The following protein samples used for Western blot analysis were prepared: (1) Proteins extracted from the salivary glands of BPH. The salivary glands of 200 newly emerged adult females of BPH were dissected and homogenized separately in 1 mL PBS. The extract was centrifuged at 12,000 × g for 5 min at 4 °C, and the supernatant used as a sample. (2) Proteins from rice leaf sheaths infested by BPH or not. Rice stems of Mudgo were individually confined within glass cylinders (diameter 4 cm, height 8 cm, with 48 small holes, diameter 0.8 mm) in which approximately 200 fifth-instar nymphs of BPH were released and, after 24 h, removed. Plants in empty glass cylinders were used as controls. The outer three leaf sheaths from three rice stems (about 0.9 g) from plants that had undergone the same treatment were harvested and merged, then ground in liquid nitrogen, homogenized in 4 ml PBS, and centrifuged at 12,000 × g for 5 min at 4 °C, after which the supernatant was concentrated to 200 μL using a YM-10 Microcon centrifugal filter device (Millipore). SDS-PAGE sample buffer (2×) was added to this extract, which was then subjected to SDS-PAGE on 12% gradient gels (ISC Bioexpress) and transferred onto a BioTrace^TM^ pure nitrocellulose blotting membrane (PALL Life Sciences). Nonspecific binding sites were blocked with 5% instant nonfat dry milk (Yili company, Hohehot municipality, Huhehaote, China), and the membrane was incubated with purified polyclonal antibody (1:200 dilution) overnight in 4 °C followed by extensive washing for 30 min with frequent changes of TBST. The antigen–antibody complexes were visualized with horseradish peroxidase–conjugated goat anti-rabbit IgG (1:5000 dilution; Multisciences, Hangzhou, China) at 37 °C for 1 h followed by extensive washing for 20 mins with frequent changes of TBST and detected with ECL substrates A and B (Multisciences) by FluorChem FC2 (Alpha Innotech).

### Ca^2+^-Binding Assays

The Ca^2+^-binding properties of NlSEF1 were confirmed by mixing purified NlSEF1 (approximately 0.75 μg/5 μL) with equal volumes of the following solutions: 0.5 mM (final concentration) EDTA or 0.015, 0.05, 0.15, or 0.5 mM CaCl_2_. Each mixture was incubated for 30 min at 25 °C, added to 10 μL of Laemmli sample buffer, and then subjected to SDS-PAGE under reducing/non-heating conditions.

### RNAi Experiment

A 598 bp fragment of *NlSEF1* and an 860 bp fragment of control gene *GFP* were amplified by PCR with primers including a T7 promoter sequence (Supplemental Table S1). The PCR products were used to synthesize dsRNA by using MEGAscript T7 High Yield Transcription Kit (Ambion, Austin, TX, USA). Third- or fifth-instar nymphs were injected at the conjunction of the prothorax and mesothorax^31^ using the FemtoJet (Eppendorf, Hamburg, Germany) microinjection device. Each nymph was injected with about 0.25 μg dsRNA of *NlSEF1* or *GFP*, or was not injected (control). The levels of *NlSEF1* transcripts in the whole body (third-instar nymphs were injected) and in the salivary gland (fifth-instar nymphs were injected) of the insect that had been injected with *NlSEF1* or *GFP* dsRNA, or kept non-injected (C-BPH), were investigated at 2, 4, 6, 8 and 10 days after injection.

### BPH Bioassays

To measure the effect of the knockdown of *NlSEF1* on mortality rates of BPH, third-instar BPH nymphs injected with *NlSEF1* or *GFP* dsRNA, or kept non-injected (C-BPH), were allowed to feed on Mudgo or TN1. The treated BPH were first allowed to recover on rice seedlings at 27 ± 1 °C with 70 ± 10% RH and 14:10 h (light/dark) photoperiod for 1 day, then the healthy ones were moved onto the tillering stage of Mudgo or TN1 rice plant for the bioassay. Stems of rice plants (one plant per pot) were individually confined within glass cylinders as stated above into which 20 third-instar nymphs were released. Each treatment had 5 replicates. The number of dead BPH nymphs in each cylinder was recorded every day.

The effect of *NlSEF1*-knockdown on BPH feeding was also investigated. A newly emerged brachypterous female adult at 3 days after the injection of *NlSEF1* or *GFP* dsRNA into fifth-instar nymphs, or no injection, was placed into a small parafilm bag (6 × 5 cm), which was then fixed onto the stem of rice variety Mudgo. The amount of excreted honeydew was weighed (to an accuracy of 0.1 mg) 24 h after the start of the experiment. Each treatment was replicated 40 times.

### Intracellular Calcium Variation Determination

The intracellular calcium variation of Mudgo plants was determined by using Fluo-3 AM (acetoxy-methyl ester of Fluo-3) as the Ca^2+^-sensitive fluorescent indicator following the method described in Maffei *et al*.^48^. Briefly, Fluo-3 AM (stock solution in dimethyl sulfoxide; Molecular Probes, Eugene, OR, USA) was diluted in 50 mM MES buffer (pH 6.0) containing 0.5 mM calcium sulfate and 2.5 μM 3-(3,4-dichlorophenyl)-1,1-dimethylurea (Sigma-Aldrich, Steinheim, Germany) to a final concentration of 5 μM. Rice leaves of Mudgo were individually confined within glass cylinders (diameter 3 cm, height 1.5 cm, with 10 small holes, diameter 0.8 mm) into which 15 newly emerged female adults of dsSEF-BPH (2 d after injection) or C-BPH were released. Infested portions of leaves were individually harvested 1, 3 and 6 h after infestation and were immediately incubated in 500 μL of 5 μM Fluo-3 AM solution as described above. Thirty minutes after incubation, leaves were mounted on a Zeiss LSM 780 confocal laser scanning microscope and were observed at 488 nm excitation wavelength. Images generated by the Zen 2010 software were analyzed by using the ImageJ software (https://imagej.nih.gov/ij). The experiment was replicated three times and the measurement for fluorescence intensity at BPH feeding sites was repeated at least 10 times.

### JA, JA-Ile, SA and Hydrogen Peroxide Analysis

Potted plants (one per pot) of Mudgo were randomly assigned to the following treatments: (1) infestation by different BPH nymph groups. Plant stems were individually confined in glass cylinders into which 20 (dsSEF-BPH) or 15 (dsGFP-BPH and C-BPH) fifth-instar nymphs (1 day after injection) were released, according to the variation of feeding ability between BPH in response to different treatments. (2) NlSEF1 treatment. Plant stems (lower part, about 2 cm long) were individually pierced 200 times with a fine needle and treated with 20 μL of either the recombinant protein NlSEF1 (0.15 μg/μL) or the purified products of the empty vector (EV), or kept non-manipulated (W). The outer three leaf sheaths of stems were harvested at different time points (Fig. 5) after the start of the treatment. JA, JA-Ile and SA levels were analyzed by high performance liquid chromatography–mass spectroscopy using labeled internal standards (^2^D_4_-SA, ^2^D_6_-JA and ^2^D_6_-JA-Ile) following the method as described in Lu *et al*.^49^. The H_2_O_2_ concentrations in the sheath were determined using Amplex-Red Hydrogen Peroxide/Peroxidase Assay Kit (Invitrogen, Carlsbad, CA, USA) as described previously by Lou and Baldwin^50^. Each treatment and time point was replicated five times.

### Data Analysis

Differences in experiments involving three treatments were analyzed by one-way ANOVAs. If the ANOVA was significant (P < 0.05), Duncan’s multiple range tests were used to detect significant differences between treatments. All tests were carried out with Statistica (SAS Institute, Inc., http://www.sas.com/).

## Additional Information

**How to cite this article**: Ye, W. *et al*. A salivary EF-hand calcium-binding protein of the brown planthopper *Nilaparvata lugens* functions as an effector for defense responses in rice. *Sci. Rep.* **7**, 40498; doi: 10.1038/srep40498 (2017).

**Publisher's note:** Springer Nature remains neutral with regard to jurisdictional claims in published maps and institutional affiliations.

## Supplementary Material

Supporting Information
