# Supplementary material for: A salivary EF-hand calcium-binding protein of the brown planthopper Nilaparvata lugens functions as an effector for defense responses in rice
Source: Sci Rep. 2017 Jan 18;7:40498. doi: 10.1038/srep40498 (PMC5241783; doi:10.1038/srep40498)
Supplement: Supporting Information [file srep40498-s1.doc]

**Supplementary information**

**A salivary EF-hand calcium-binding protein of the brown planthopper *Nilaparvata lugens* functions as an effector for defense responses in rice**

Wenfeng Ye, Haixin Yu, Jiamei Zeng, Yukun Jian, Rui Ji, Hongdan Chen, Yonggen Lou*

State Key Laboratory of Rice Biology, Institute of Insect Science, Zhejiang University, Hangzhou 310058, China

**Supplemental Figures and Tables**

**Supplemental Figure S1.** Secondary structure analysis of NlSEF1.

PRED represents the predicted secondary structure of NlSEF1. Rows “C” and “H” indicate the predicted random coils and α-helixes, respectively. Sequences of the two EF-hand domains are boxed in yellow shading.

**Supplemental Figure S2.** Sequence alignment of homologous EF-hand proteins.

Selected sequences accession numbers: Nv [(](http://blast.ncbi.nlm.nih.gov/Blast.cgi#alnHdr_156538673)*Nasonia vitripennis*,[XP_001607749.1](http://www.ncbi.nlm.nih.gov/protein/156538673?report=genbank&log$=prottop&blast_rank=3&RID=JD24TZDR016)), Fa (*[Fopius arisanus](http://blast.ncbi.nlm.nih.gov/Blast.cgi#alnHdr_755977452)*, [XP_011308288.1](http://www.ncbi.nlm.nih.gov/protein/755977452?report=genbank&log$=prottop&blast_rank=4&RID=JD24TZDR016)), Bi (*[Bombus impatiens](http://blast.ncbi.nlm.nih.gov/Blast.cgi#alnHdr_350419780)*, [XP_003492298.1](http://www.ncbi.nlm.nih.gov/protein/350419780?report=genbank&log$=prottop&blast_rank=5&RID=JD24TZDR016)), Si (*[Solenopsis invicta](http://blast.ncbi.nlm.nih.gov/Blast.cgi#alnHdr_751211167)*, [XP_011158313.1](http://www.ncbi.nlm.nih.gov/protein/751211167?report=genbank&log$=prottop&blast_rank=1&RID=JD24TZDR016)), Tc (*[Tribolium castaneum](http://blast.ncbi.nlm.nih.gov/Blast.cgi#alnHdr_91082581)*, [XP_967080.1](http://www.ncbi.nlm.nih.gov/protein/91082581?report=genbank&log$=prottop&blast_rank=2&RID=JD24TZDR016)), Pc (*[Pediculus humanus corporis](http://blast.ncbi.nlm.nih.gov/Blast.cgi#alnHdr_242018051)*, [XP_002429496.1](http://www.ncbi.nlm.nih.gov/protein/242018051?report=genbank&log$=prottop&blast_rank=11&RID=JD24TZDR016)), Nl (*Nilaparvata lugens*, NlSEF1). The predicted signal peptide is underlined. “*” under sequences indicates identical amino acids, “:” and “.” means conserved and semiconserved amino acid sites, respectively. Sequences of the two EF-hand domains are marked in red letters.

**Supplemental Figure S3.** The silencing efficiency of *NlSEF1* by RNAi.

A, Mean transcript levels (+SE, n = 3) of *NlSEF1* in whole bodies on different days after third-instar BPH nymphs had been injected with dsRNA of *NlSEF1* (*dsSEF*) or GFP (*dsGFP*), or kept non-injected (C-BPH).

B, Mean transcript levels (+SE, n = 3) of *NlSEF1* in salivary gland of newly emerged brachypterous female adults 3 days after they (fifth-instar nymphs) had been treated as stated above.

Letters indicate significant differences among different treatments (p<0.05, Duncan’s multiple range test).

**Supplemental Figure S4.** The growth phenotype of BPH nymphs. No phenotypic differences were observed 5 days after nymphs had been injected with either *dsGFP* (left) or *dsSEF* (right).

**Supplemental Table S1.** Primers used for qRT-PCR and PCR.

**Supplemental Figures**

**
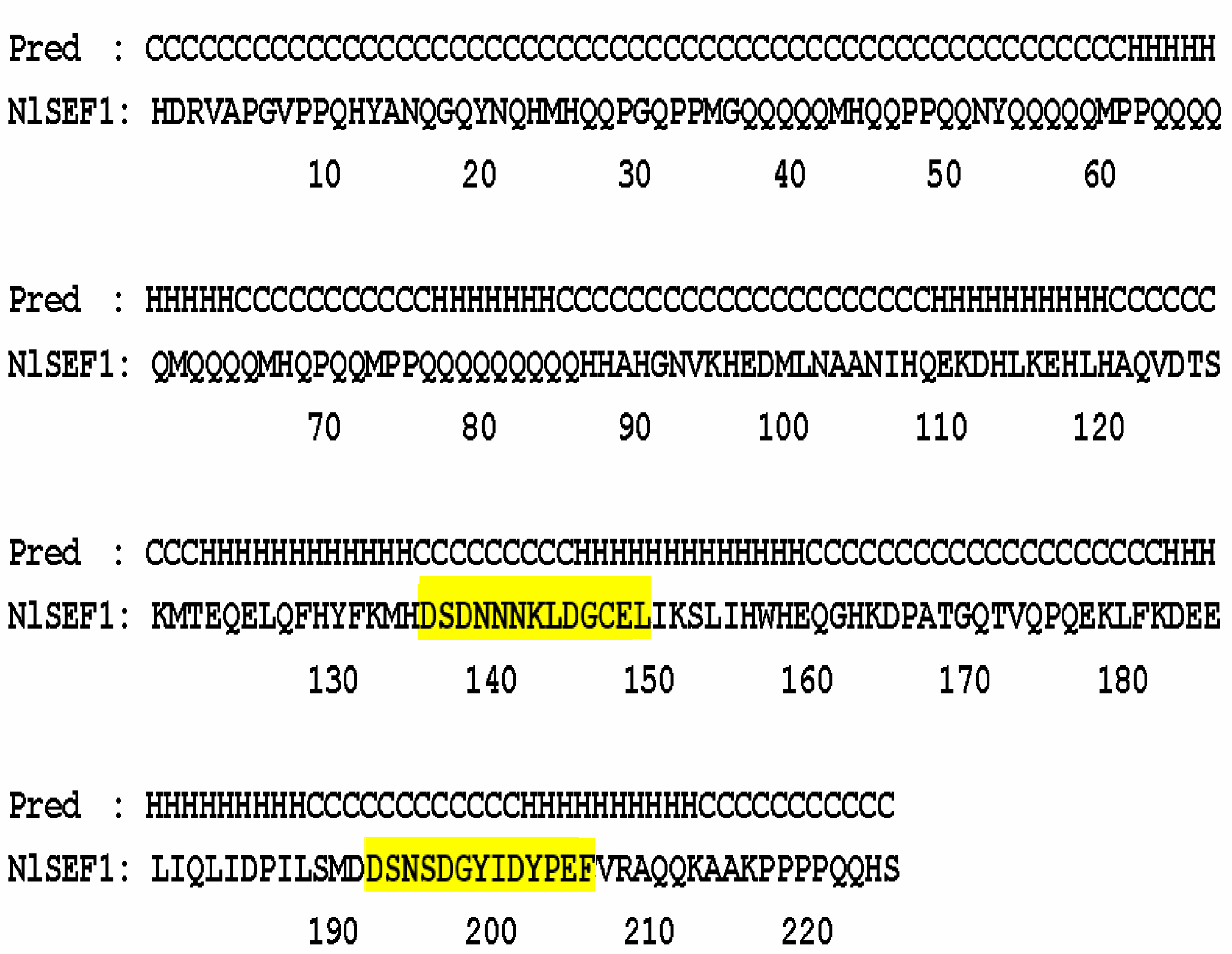
**

**Supplemental Figure S1.** Secondary structure analysis of NlSEF1.

PRED represents the predicted secondary structure of NlSEF1. Rows“C” and “H” indicate the predicted random coils and α-helixes, respectively. Sequences of the two EF-hand domains are boxed in yellow shading.


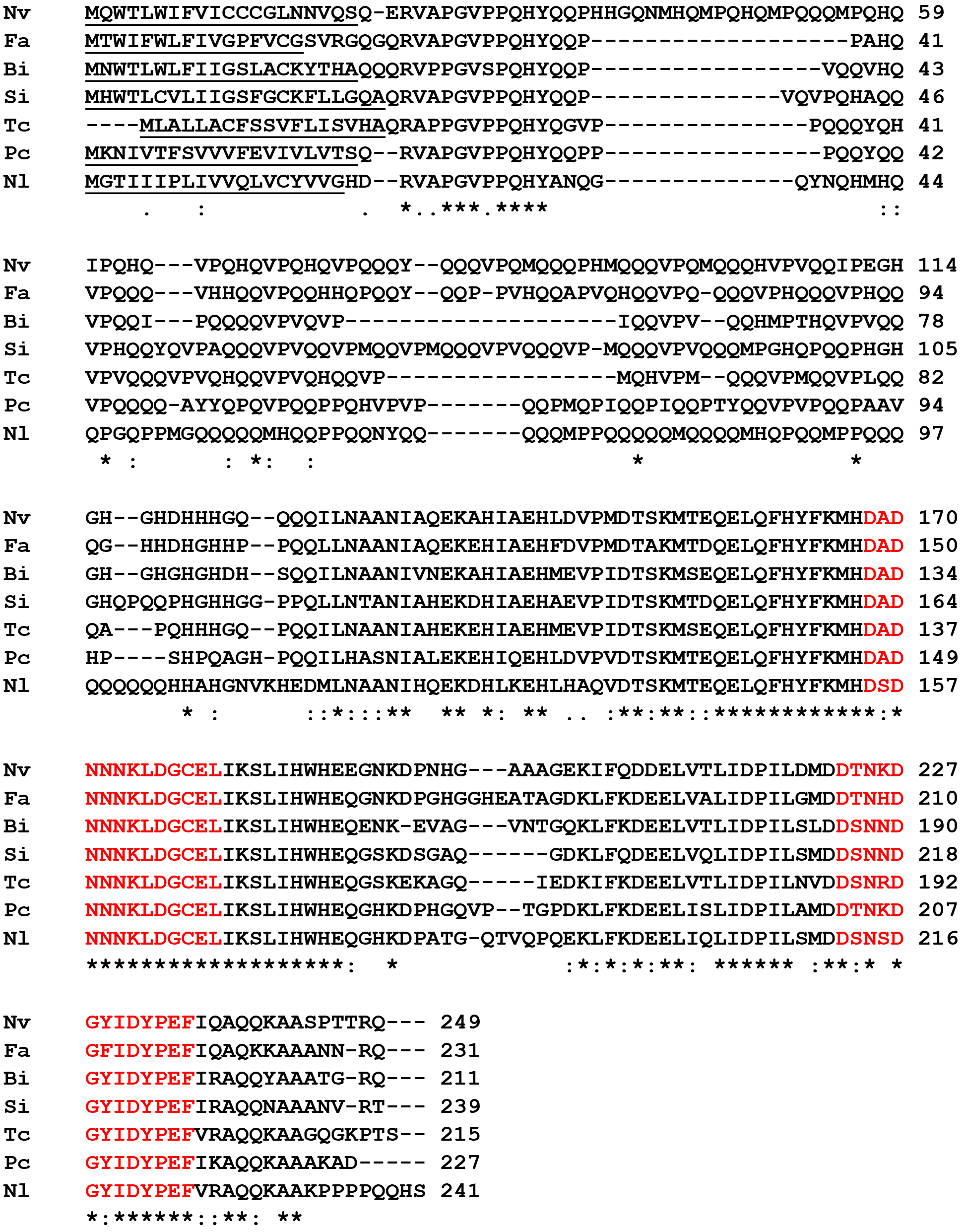


**Supplemental Figure S2.** Sequence alignment of homologous EF-hand proteins.

Selected sequences accession numbers: Nv [(](http://blast.ncbi.nlm.nih.gov/Blast.cgi#alnHdr_156538673)*Nasonia vitripennis*,[XP_001607749.1](http://www.ncbi.nlm.nih.gov/protein/156538673?report=genbank&log$=prottop&blast_rank=3&RID=JD24TZDR016)), Fa (*[Fopius arisanus](http://blast.ncbi.nlm.nih.gov/Blast.cgi#alnHdr_755977452)*,[XP_011308288.1](http://www.ncbi.nlm.nih.gov/protein/755977452?report=genbank&log$=prottop&blast_rank=4&RID=JD24TZDR016)), Bi (*[Bombus impatiens](http://blast.ncbi.nlm.nih.gov/Blast.cgi#alnHdr_350419780)*, [XP_003492298.1](http://www.ncbi.nlm.nih.gov/protein/350419780?report=genbank&log$=prottop&blast_rank=5&RID=JD24TZDR016)), Si (*[Solenopsis invicta](http://blast.ncbi.nlm.nih.gov/Blast.cgi#alnHdr_751211167)*, [XP_011158313.1](http://www.ncbi.nlm.nih.gov/protein/751211167?report=genbank&log$=prottop&blast_rank=1&RID=JD24TZDR016)), Tc (*[Tribolium castaneum](http://blast.ncbi.nlm.nih.gov/Blast.cgi#alnHdr_91082581)*, [XP_967080.1](http://www.ncbi.nlm.nih.gov/protein/91082581?report=genbank&log$=prottop&blast_rank=2&RID=JD24TZDR016)), Pc (*[Pediculus humanus corporis](http://blast.ncbi.nlm.nih.gov/Blast.cgi#alnHdr_242018051)*, [XP_002429496.1](http://www.ncbi.nlm.nih.gov/protein/242018051?report=genbank&log$=prottop&blast_rank=11&RID=JD24TZDR016)), Nl (*Nilaparvata lugens*, NlSEF1). The predicted signal peptide is underlined. “*” under sequences indicates identical amino acids, “:” and “.” means conserved and semiconserved amino acid sites, respectively. Sequences of the two EF-hand domains are marked in red letters.


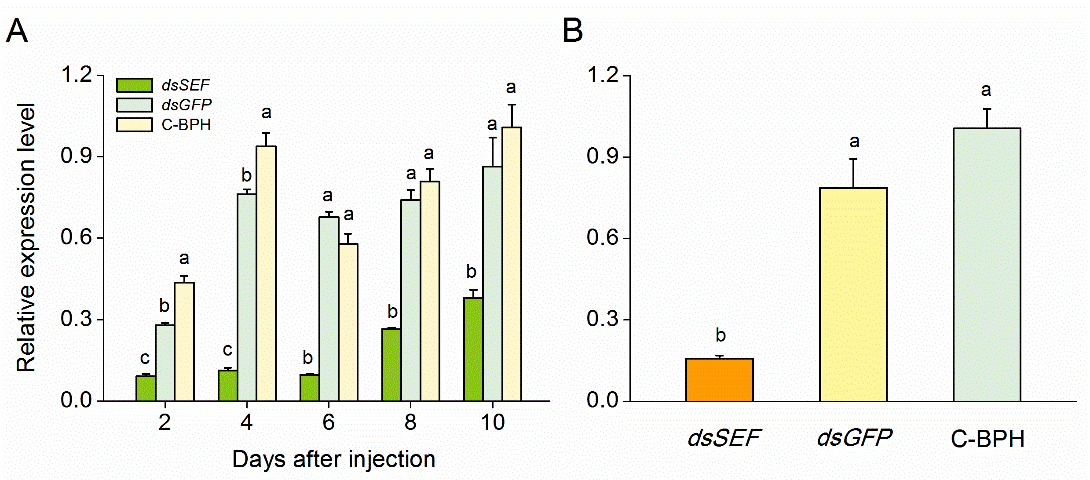


**Supplemental Figure S3.** The silencing efficiency of *NlSEF1* by RNAi.

A, Mean transcript levels (+SE, n = 3) of *NlSEF1* in whole bodies on different days after third-instar BPH nymphs had been injected with dsRNA of *NlSEF1* (*dsSEF*) or GFP (*dsGFP*), or kept non-injected (C-BPH).

B, Mean transcript levels (+SE, n = 3) of *NlSEF1* in salivary gland of newly emerged brachypterous female adults 3 days after they (fifth-instar nymphs) had been treated as stated above.

Letters indicate significant differences among different treatments (p<0.05, Duncan’s multiple range test).


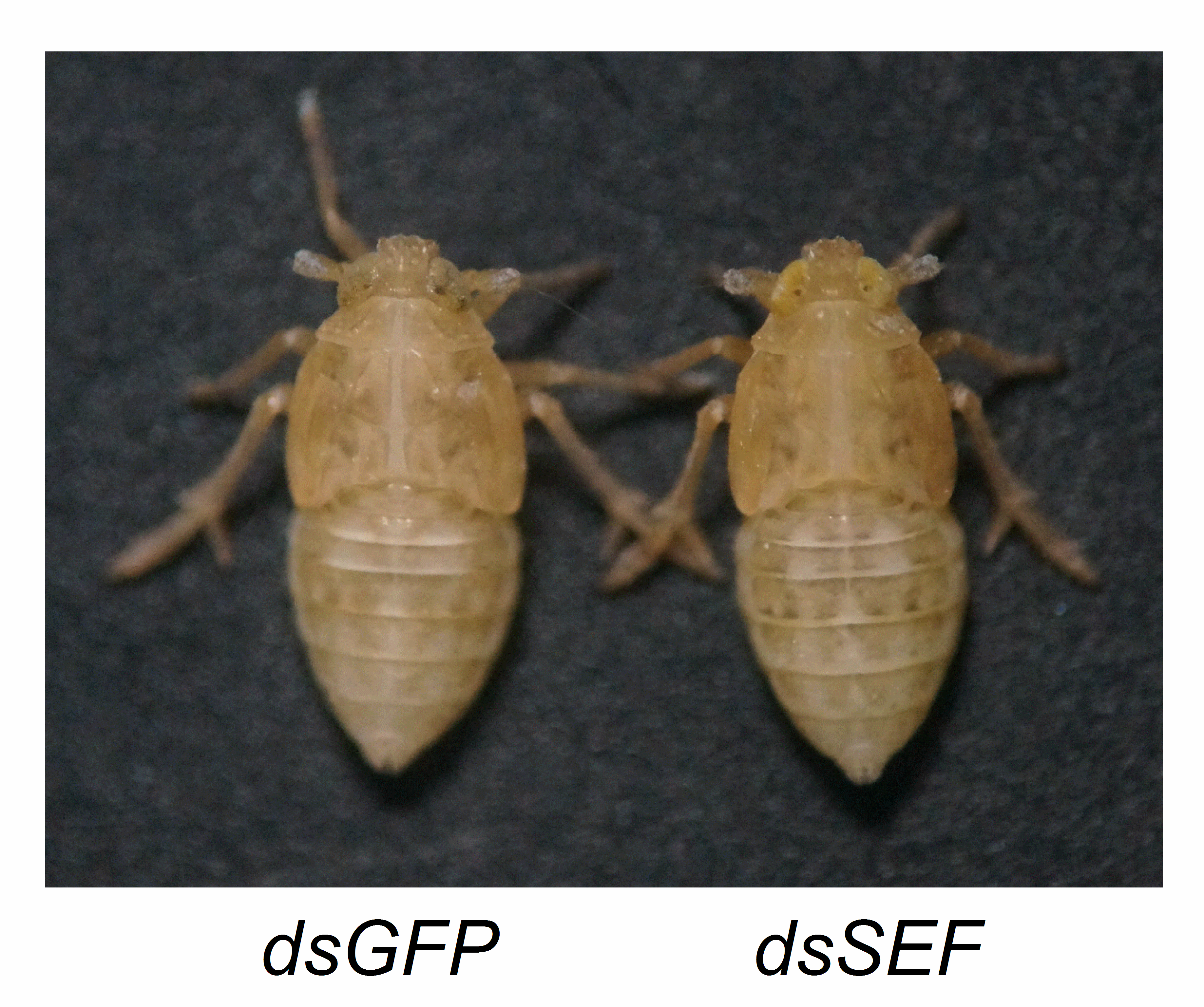


**Supplemental Figure S4.** The growth phenotype of BPH nymphs. No phenotypic differences were observed 5 days after nymphs had been injected with either *dsGFP* (left) or *dsSEF* (right).

**Supplemental Table S1.** Primers used for qRT-PCR and PCR.

| **Gene** | **Description** | **Forward primer (5'-…-3')** | **Reverse primer (5'…-3')** |  |  |  |  |  |  |  |  |  |  |  |
| --- | --- | --- | --- | --- | --- | --- | --- | --- | --- | --- | --- | --- | --- | --- |
| *NlSEF1* | QPCR | TGGTGGAACCTTGGAACAG | CACGTTGCAGGTTATGACATC |  |  |  |  |  |  |  |  |  |  |  |
| *18S rRNA* | QPCR | CGCTACTACCGATTGAA | GGAAACCTTGTTACGACTT |  |  |  |  |  |  |  |  |  |  |  |
| *NlSEF1* | Cloning | GTCCTGCATGAATCCTGA | CATCGAAATCTGAGTCGTA |  |  |  |  |  |  |  |  |  |  |  |
| *NlSEF1* | Protein expression | CGCGGATCCCATGATAGGGTGGCACCA | CGCTCGAGCAGTTTCACGAGTGCTGCT |  |  |  |  |  |  |  |  |  |  |  |
| *NlSEF1* | dsRNA synthesis | GGATCCTAATACGACTCACTATAGGGA  CATGCTCAACGCAGCCAA | GGATCCTAATACGACTCACTATAGGGT  AACCGTCGCTGTTGCTGT |  |  |  |  |  |  |  |  |  |  |  |
| *GFP* | dsRNA synthesis | GGATCCTAATACGACTCACTATAGGAA  GGGCGAGGAGCTGTTCACCG | GGATCCTAATACGACTCACTATAGGCA  GCAGGACCATGTGATCGCGC |  |  |  |  |  |  |  |  |  |  |  |
